# Modeling the Cost-Effectiveness of Adjuvant Chemotherapy for Stage III Colon Cancer in South African Public Hospitals

**DOI:** 10.1200/GO.21.00279

**Published:** 2021-12-22

**Authors:** Yoanna Pumpalova, Alexandra M. Rogers, Sarah Xinhui Tan, Candice-lee Herbst, Paul Ruff, Alfred I. Neugut, Chin Hur

**Affiliations:** ^1^ Department of Medicine, Vagelos College of Physicians and Surgeons, Columbia University, New York, NY; ^2^ Department of Internal Medicine, Faculty of Health Sciences, University of the Witwatersrand, Johannesburg, South Africa; ^3^ Noncommunicable Diseases Research Division, Wits Health Consortium (PTY) Ltd, Johannesburg, South Africa; ^4^ SAMRC/Wits Developmental Pathways to Health Research Unit, Department of Paediatrics, Faculty of the Health Sciences, University of the Witwatersrand, Johannesburg, South Africa; ^5^ Division of Medical Oncology, Department of Medicine, Faculty of Health Sciences, University of the Witwatersrand, Johannesburg, South Africa; ^6^ Herbert Irving Comprehensive Cancer Center, Vagelos College of Physicians and Surgeons, Columbia University, New York, NY; ^7^ Department of Epidemiology, Mailman School of Public Health, Columbia University, New York, NY

## Abstract

**PURPOSE:**

Cancer incidence is rising in low- and middle-income countries, where resource constraints often complicate therapeutic decisions. Here, we perform a cost-effectiveness analysis to identify the optimal adjuvant chemotherapy strategy for patients with stage III colon cancer treated in South African (ZA) public hospitals.

**METHODS:**

A decision-analytic Markov model was developed to compare lifetime costs and outcomes for patients with stage III colon cancer treated with six adjuvant chemotherapy regimens in ZA public hospitals: fluorouracil, leucovorin, and oxaliplatin for 3 and 6 months; capecitabine and oxaliplatin (CAPOX) for 3 and 6 months; capecitabine for 6 months; and fluorouracil/leucovorin for 6 months. Transition probabilities were derived from clinical trials to estimate risks of toxicity, disease recurrence, and survival. Societal costs and utilities were obtained from literature. The primary outcome was the incremental cost-effectiveness ratio in international dollars (I$) per disability-adjusted life-year (DALY) averted, compared with no therapy, at a willingness-to-pay (WTP) threshold of I$13,006.56.

**RESULTS:**

CAPOX for 3 months was cost-effective (I$5,381.17 and 5.74 DALYs averted) compared with no adjuvant chemotherapy. Fluorouracil, leucovorin, and oxaliplatin for 6 months was on the efficiency frontier with 5.91 DALYs averted but, with an incremental cost-effectiveness ratio of I$99,021.36/DALY averted, exceeded the WTP threshold.

**CONCLUSION:**

In ZA public hospitals, CAPOX for 3 months is the cost-effective adjuvant treatment for stage III colon cancer. The optimal strategy in other settings may change according to local WTP thresholds. Decision analytic tools can play a vital role in selecting cost-effective cancer therapeutics in resource-constrained settings.

## INTRODUCTION

Cancer incidence is rising in low- and middle-income countries (LMICs), where health systems are often ill equipped to provide costly cancer care.^1^ By 2040, new cancer cases in sub-Saharan Africa (SSA) are predicted to increase by 95%; publicly funded health systems in SSA must adapt now to meet this demand.^1^ Cost-effectiveness analyses can provide critical evidence to inform which cancer therapeutic strategies resource-constrained health systems should fund. In this study, we perform a cost-effectiveness analysis to identify the optimal adjuvant chemotherapy strategy for adults treated for stage III colon cancer in South African (ZA) public hospitals.

**CONTEXT**

**Key Objective**
What is the cost-effective adjuvant chemotherapy regimen for patients with stage III colon cancer treated in South African public hospitals?
**Knowledge Generated**
In our base case, capecitabine and oxaliplatin (CAPOX) for 3 months was the cost-effective strategy for patients with stage III colon cancer, with a lifetime cost of 5,381.17 international dollars (I$) compared with I$9,959.24 for no adjuvant chemotherapy (surgery alone). Fluorouracil, leucovorin, and oxaliplatin for 6 months was on the efficiency frontier, but far exceeded the willingness-to-pay threshold (set at the 2020 South African Gross Domestic Product). In a scenario analysis of patients with stage III colon cancer stratified by colon cancer recurrence risk, CAPOX for 6 months was cost-effective for high-risk patients.
**Relevance**
We show that adjuvant chemotherapy with CAPOX for 3 months is cost-effective for treating patients with stage III colon cancer in South African public hospitals and should be the preferred regimen.

Although historically rare in LMICs, colon and rectal cancer (CRC) incidence increases with economic development, rendering CRC treatment a useful case study for modeling cancer care costs.^2^ ZA is an upper middle-income country in SSA and CRC incidence increased by 30% in ZA males between 2000 and 2014, a trend similar to that expected across SSA.^3^ Approximately half of CRCs in ZA occur in the colon, and up to 50% of patients present with locally advanced (stage III) disease and have an indication for adjuvant chemotherapy.^4,5^

To meet this increasing demand for adjuvant chemotherapy, health systems can develop disease-specific treatment pathways, encouraging use of the most cost-effective agents available. Such treatment pathways exist in high-income countries (HICs) with national health systems and have been associated with significant cost-savings to the patient and health system.^6^ In 2018, Herbst et al^7^ compared the health care system costs of chemotherapy regimens for colon cancer in ZA public hospitals. Building upon this work, we now evaluate the lifetime costs and effectiveness, in comparison to surgery alone, of adjuvant chemotherapy regimens for male and female adult patients treated for stage III colon cancer in ZA public hospitals.

## METHODS

### Study Design

We developed a decision-analytic Markov model from a societal perspective to assess the lifetime costs and health outcomes associated with six adjuvant chemotherapy regimens, with no adjuvant therapy (surgery alone) as the comparator. The societal perspective accounts for direct medical costs to the health care system associated with treatment and surveillance and indirect costs of care incurred by the patient and a caregiver (Table 1). The tested regimens were fluorouracil, leucovorin, and oxaliplatin (FOLFOX) for 3 and 6 months; capecitabine and oxaliplatin (CAPOX) for 3 and 6 months; capecitabine for 6 months; and fluorouracil and leucovorin (5-Fluorouracil and leucovorin [FU/LV]; Mayo regimen) for 6 months. The model was developed and analyzed using TreeAge Pro Healthcare 2021.

**TABLE 1:**
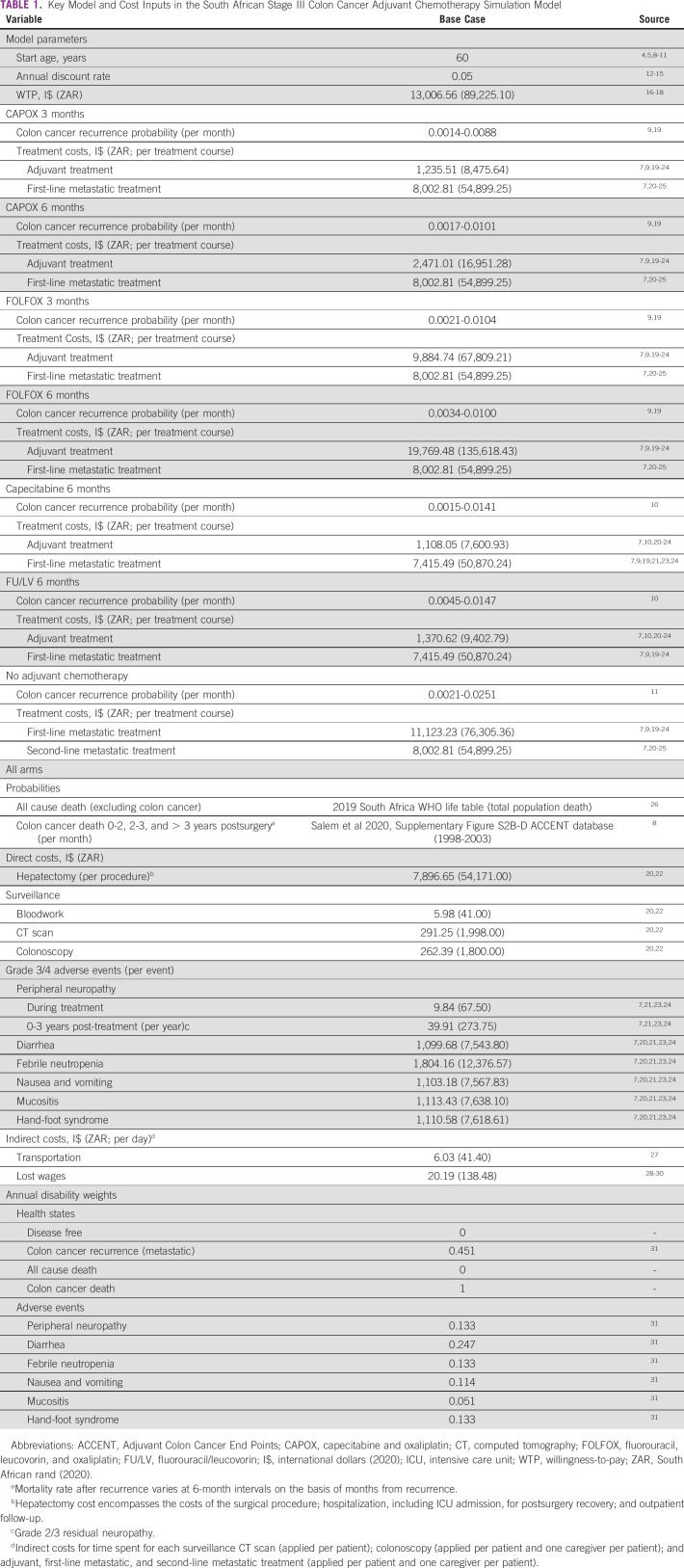
Key Model and Cost Inputs in the South African Stage III Colon Cancer Adjuvant Chemotherapy Simulation Model

### Model Overview

**FIG 1:**
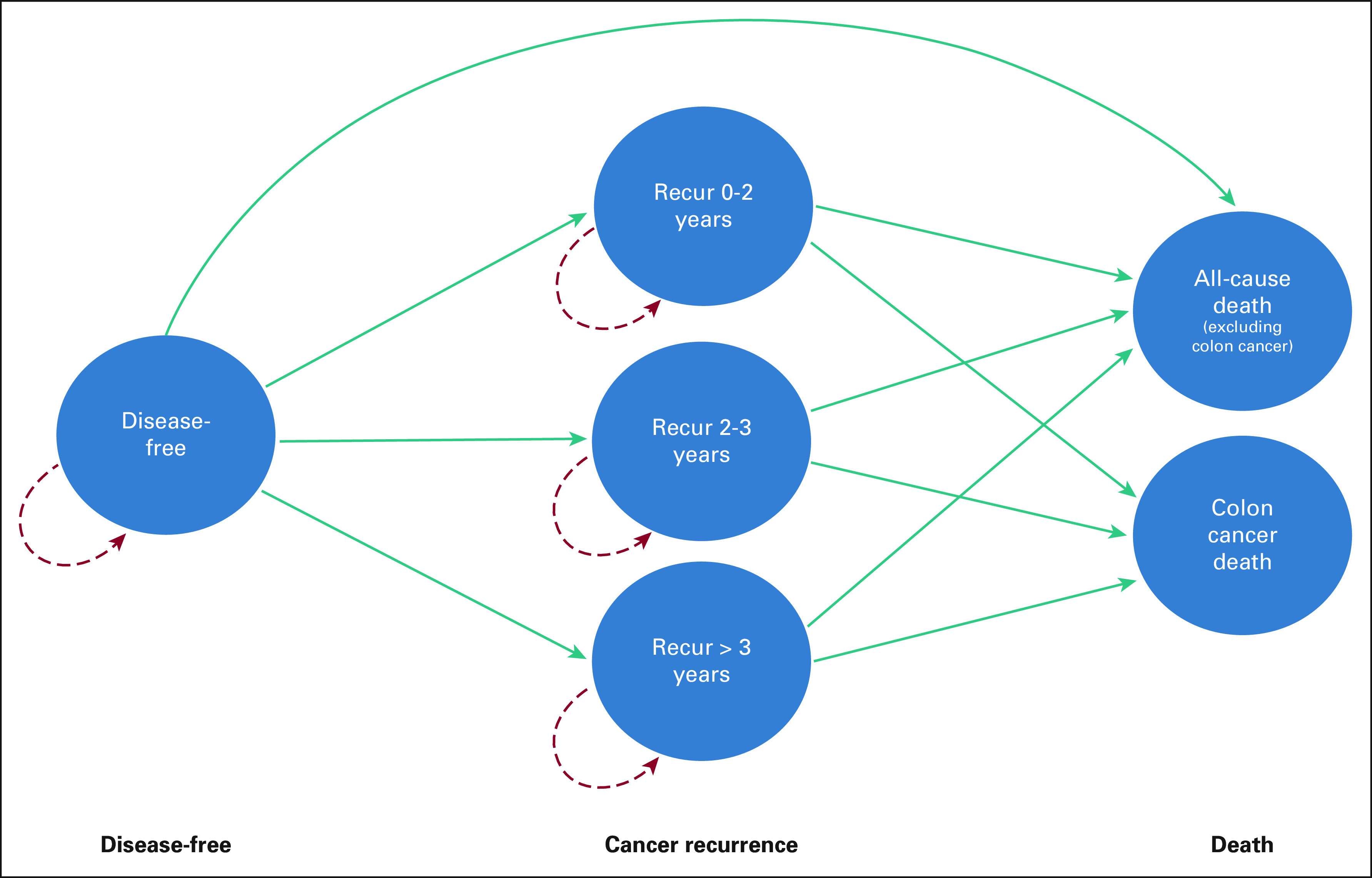
Markov model state transition diagram. Structure of the decision-analytic Markov tree constructed and used to evaluate the cost-effectiveness of adjuvant treatments for patients with stage III colon cancer in the South African public health care setting, showing the cohort entering the model in the disease-free state after surgical resection, transitioning to various cancer recurrence states, and eventually ending in one of two death states: death from all other causes excluding colon cancer or death from colon cancer.

Our model simulated the disease progression following curative resection in a hypothetical cohort of 60-year-old male and female patients with stage III colon cancer. The hypothetical cohort start age of 60 years was chosen to reflect the average age of patients with CRC in the South African National Cancer Registry^32^ and is comparable to the median age of participants in the randomized controlled clinical trials (RCTs) used to derive model inputs (Data Supplement).^8-11^ All patients entered the model in the disease-free state (defined as free of colon cancer recurrence) and either remained disease-free or transitioned to colon cancer recurrence, death from colon cancer, or death from all other causes excluding colon cancer (Fig 1). Patients in the disease-free state were considered cured of colon cancer if they remained in that state for more than 96 months (8 years).^33^

All colon cancer recurrences were assumed to be clinical distant metastases. Individuals who recurred transitioned to a recurrence health state, according to when they recurred relative to the model start (0-2 years, 2-3 years, or > 3 years). On the basis of literature review and local expert opinion (P.R. and C.-L.H.), we assumed that one third of recurrences were liver-only and that one third of these cases proceeded to curative-intent hepatectomy.^34^ On the basis of local practices, our model assumed no use of biologic agents in the metastatic setting and a maximum of two lines of chemotherapy per patient (Data Supplement).^7^ We derived toxicity estimates from RCTs for the six most commonly reported treatment-related adverse events (TRAEs) in the adjuvant and metastatic settings (Table 1; Data Supplement).^10,25,35,36^ The model ran for 300 months, or until age 85 years, at which point the mortality rate in ZA approximates 100%.^9,11,26^ The model cycle length was 1 month, and a half-cycle correction was applied.

### Model Inputs

Table 1 provides a summary of the key model inputs. All transition probabilities were derived from published literature. Recurrence rates were estimated from disease-free survival (DFS) and recurrence-free curves from RCTs (Data Supplement).^9,11,19^ We used Engauge Digitizer Software^37^ to trace relevant Kaplan-Meier curves and RStudio 1.3.1073 software^38^ to fit spline functions to the data to calculate monthly recurrence probabilities. Survival probabilities after recurrence were obtained from a pooled analysis of patients in the Adjuvant Colon Cancer End Points (ACCENT) database; probabilities of death from colon cancer were applied on the basis of time of recurrence relative to surgery and length of time in the recurrence state, regardless of initial treatment received.^8,39^ Death from all causes excluding colon cancer was accounted for by applying age-specific background mortality rates obtained from the WHO 2019 life tables for ZA from age 60 to 85 years.^26^

Direct medical costs were obtained from the ZA Department of Health's 2020 Uniform Patient Fee Schedule and the South African Medicine Price Registry's Database of Medicine Prices and included the drug, personnel, administration, antiemetics, and bloodwork costs for each regimen, as well as drug and hospitalization costs for each TRAE (Data Supplement).^20,21^ Costs of surveillance were applied to all patients in the disease-free state (Data Supplement). The cost of an open hepatectomy after recurrence was based on key informant interviews with surgeons in ZA.^20,22,40^ All costs were converted from 2020 South African rand (ZAR) to international dollars (I$) using the 2020 purchasing power parity (PPP; conversion factor: ZAR/6.86 PPP = I$).^41^ All treatment regimens included in our model are available in the ZA public health care sector. As the majority of patients served by the ZA public health care sector qualify for free health care on the basis of their income level, direct costs were assumed to be incurred by the government.

Indirect costs were calculated for the patient and one caregiver and included transportation to and from the clinic and wages lost because of treatment time or surveillance. The average round trip cost using public transportation was estimated at I$6.03.^27^ For every treatment visit, we assumed a day of lost wages (8 hours × I$2.52, on the basis of the maximum annual income qualifying patients for free health care in ZA).^28-30^

Quality of life utility estimates were obtained from literature^31^ and expressed in terms of disability-adjusted life-years (DALYs). All utility values ranged from 0 (no disability) to 1 (death from colon cancer). An annual global discounting rate of 5% was applied to all costs and utilities, as recommended by the National Department of Health and used in previous cost-effectiveness analyses done in ZA.^12,14,42^

### Outcomes

Our primary outcomes were death from colon cancer and disability because of colon cancer recurrence and/or TRAEs. We report our primary results as the incremental cost-effectiveness ratio (ICER) in I$ per DALY averted. DALYs were calculated for each treatment strategy by summing the years of life lost and years lived with disability.^43^ Model results and recommendations reference a base case willingness-to-pay (WTP) threshold equal to the 2020 gross domestic product (GDP) per capita of ZA (I$13,006.56).^16-18^ Overall survival (OS) is reported as undiscounted and unadjusted life-years. All other analyses of cost-effectiveness are discounted and adjusted for disability, without age-weighting.^13,14,44^

A secondary output of the model was the societal net monetary benefit (NMB) for each strategy, calculated using the formula:

NMB=WTP×Effectiveness−Cost.

### Sensitivity and Scenario Analyses

In deterministic sensitivity analyses, we varied key model variables one at a time according to ranges reported in the literature and common practices in economic evaluations.^45-47^ In a probabilistic sensitivity analysis (PSA) with 100,000 random iterations, we varied all key variables simultaneously, as well as the WTP threshold from one half (I$6,503.29) to three times (I$39,019.72) the ZA GDP per capita.^18,42,44,48,49^ Table 2 summarizes the variables, distributions, and ranges used in these sensitivity analyses.

**TABLE 2:**
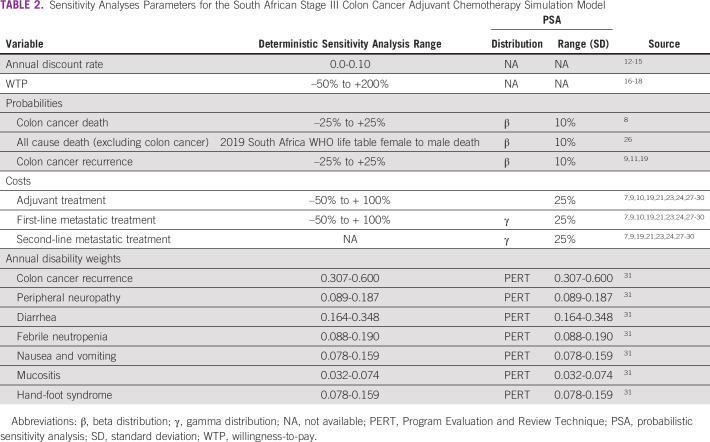
Sensitivity Analyses Parameters for the South African Stage III Colon Cancer Adjuvant Chemotherapy Simulation Model

Finally, we performed a risk-stratified scenario analysis of the cost-effectiveness of the four oxaliplatin-based chemotherapy regimens evaluated in the base case (CAPOX and FOLFOX for 3 and 6 months). Risk-stratification definitions and risk-specific recurrence probabilities were derived from the International Duration Evaluation of Adjuvant Therapy (IDEA) collaboration (Data Supplement).^9,50^

### Role of the Funding Source

The funders of the study had no role in study design, data collection, data analysis, data interpretation, or writing of the report. All authors had full access to all the data in the study and had final responsibility for the decision to submit for publication.

### Ethical Approval

Ethical approval for obtaining the cost data associated with adjuvant colon cancer treatment was obtained through the Human Research Ethics Committee at the University of Witwatersrand (M1409809).

## RESULTS

### Base Case Cost-Effectiveness Analysis

The base case results are presented in Table 3, including the total cost, OS, and DALYs averted for each strategy. Model DFS outputs closely approximated those reported in RCTs (Data Supplement). For a hypothetical cohort of 60-year-old patients with stage III colon cancer treated in ZA public hospitals, the cost-effective strategy was CAPOX for 3 months (CAPOX 3MO), with a lifetime cost of I$5,381.17 and 5.74 DALYs averted per patient compared with no adjuvant chemotherapy. The lifetime cost of no adjuvant chemotherapy was I$9,959.24 per patient, significantly higher than that for CAPOX 3MO. All strategies had a positive societal NMB except for no adjuvant chemotherapy, indicating that all treatments are more cost-effective than no adjuvant chemotherapy. CAPOX 3MO had the highest NMB of I$69,230.90.

**TABLE 3:**
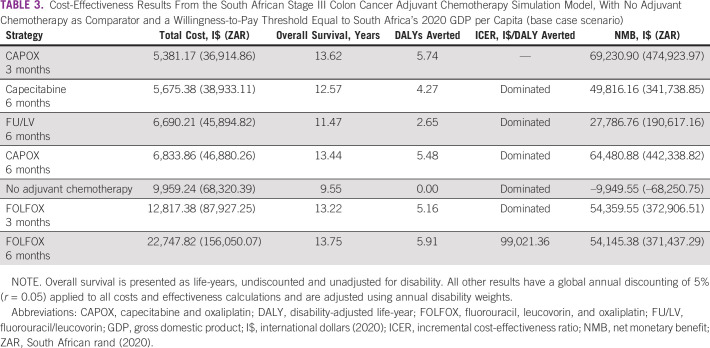
Cost-Effectiveness Results From the South African Stage III Colon Cancer Adjuvant Chemotherapy Simulation Model, With No Adjuvant Chemotherapy as Comparator and a Willingness-to-Pay Threshold Equal to South Africa's 2020 GDP per Capita (base case scenario)

**FIG 2:**
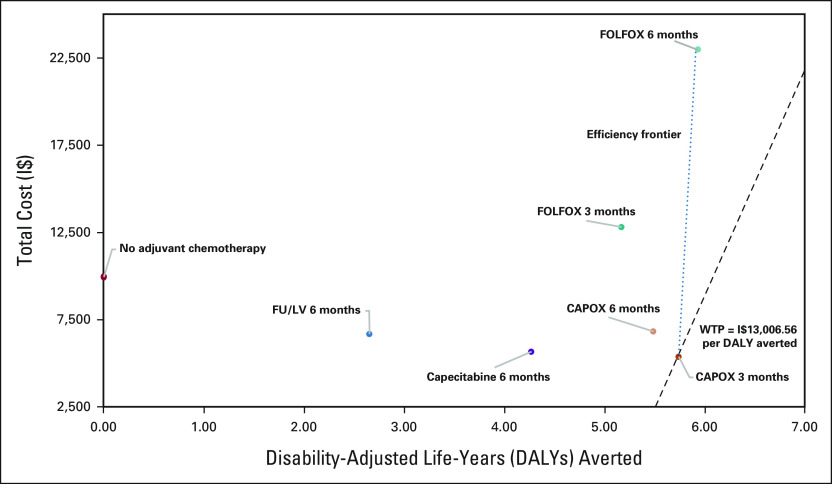
Base case cost-effectiveness analysis of six adjuvant therapy regimens compared with receiving no adjuvant chemotherapy. The figure shows that only two treatment strategies, CAPOX for 3 months and FOLFOX for 6 months, are undominated and lie on the efficiency frontier (blue dashed line), and only CAPOX for 3 months is cost-effective. FOLFOX for 6 months is not cost-effective as the ICER exceeds the WTP threshold (gray dashed line). CAPOX, capecitabine and oxaliplatin; DALY, disability-adjusted life-year; FOLFOX, fluorouracil, leucovorin, and oxaliplatin; FU/LV, fluorouracil/leucovorin; I$, international dollars (2020); ICER, incremental cost-effectiveness ratio; WTP, willingness-to-pay. Adjuvant CAPOX for 3 months is cost-effective for surgically resected stage III colon cancer in South Africa

FOLFOX for 6 months (FOLFOX 6MO) was on the efficiency frontier, with a slightly higher effectiveness than the optimal strategy at an additional 0.17 DALYs averted but a significantly higher lifetime cost (I$22,747.82). The ICER of I$99,021.36/DALY averted for FOLFOX 6MO was above the WTP threshold and the NMB ($54,145.38) was lower than that for CAPOX 3MO. All other strategies were dominated (Fig 2).

The unadjusted OS for patients treated with adjuvant chemotherapy ranged from 11.47 to 13.75 years compared with 9.55 years for no adjuvant chemotherapy. Our model predicted that oxaliplatin-based adjuvant chemotherapy is more effective than single-agent FU/LV or capecitabine (OS 13.22-13.75 years *v* 11.47-12.57 years) and that CAPOX 3MO was slightly more effective than CAPOX for 6 months (CAPOX 6MO) (OS 13.62 *v* 13.44 years), whereas FOLFOX 6MO was more effective than FOLFOX for 3 months (FOLFOX 3MO; OS 13.75 *v* 13.44 years).

### Sensitivity Analyses

**FIG 3:**
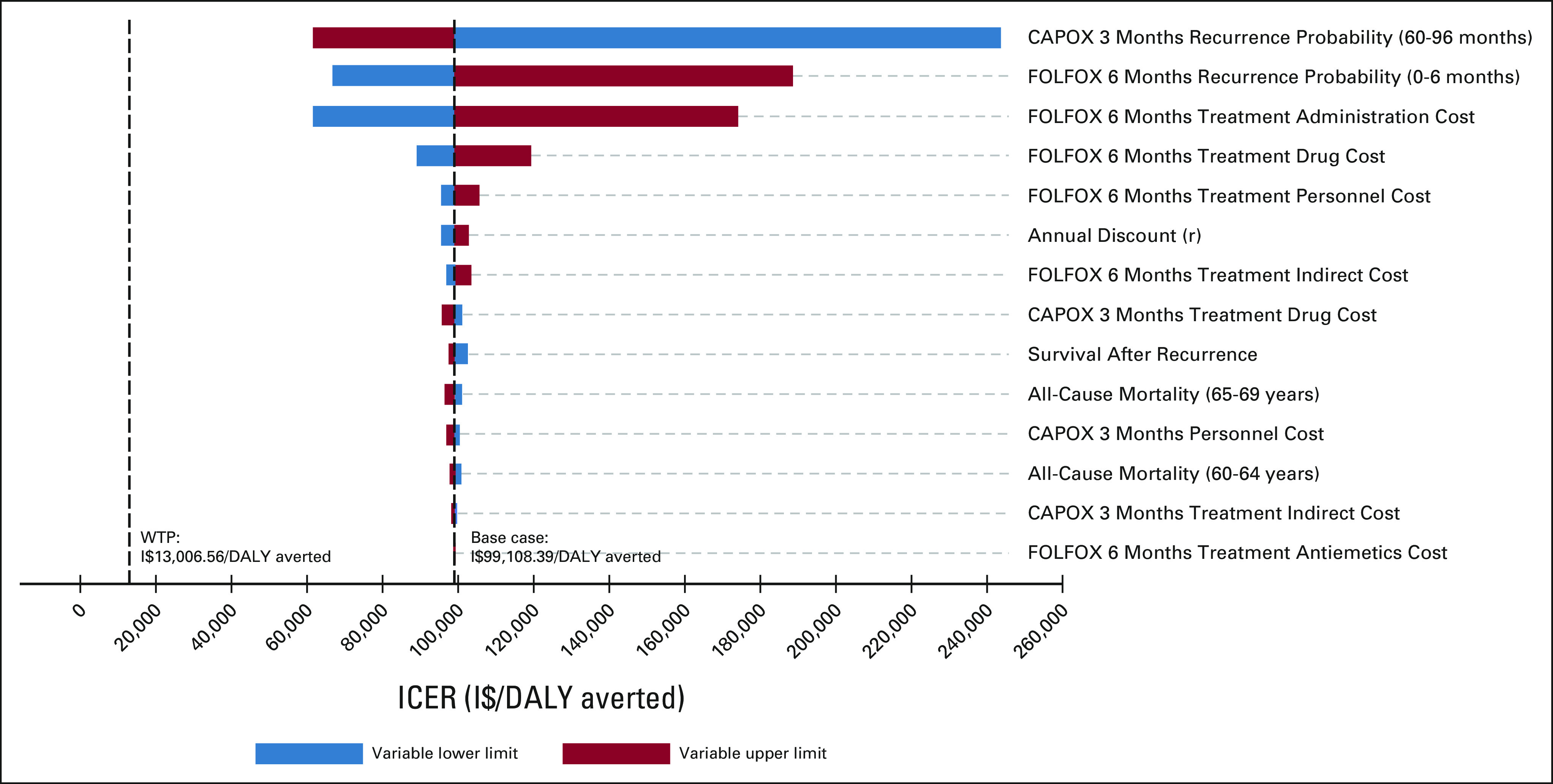
One-way deterministic sensitivity analysis. This tornado diagram illustrates the results of a one-way sensitivity analysis of key model inputs. The plot shows the impact of varying these parameters on the ICER of FOLFOX for 6 months, the only other strategy on the efficiency frontier, in comparison to CAPOX for 3 months, the optimal strategy. CAPOX, capecitabine and oxaliplatin; DALY, disability-adjusted life-year; FOLFOX, fluorouracil, leucovorin, and oxaliplatin; I$, international dollars (2020); ICER, incremental cost-effectiveness ratio; r, rate; WTP, willingness-to-pay.

One-way deterministic sensitivity analyses indicated that our base case results were robust to uncertainty in model parameters. Figure 3 illustrates these results for the two strategies on the efficiency frontier, CAPOX 3MO and FOLFOX 6MO. The probability of colon cancer recurrence after adjuvant CAPOX 3MO had the greatest effect on model outcomes. However, no parameter affected the recommendations of the model as CAPOX 3MO remained optimal.

The higher direct costs of FOLFOX 6MO compared with CAPOX 3MO were driven by longer duration of treatment (6 *v* 3 months) and higher administration costs associated with FOLFOX (ie, placement of port-a-cath and use of continuous infusion pump; Data Supplement). Thus, we conducted an additional one-way sensitivity analysis isolating the administration cost of FOLFOX 6MO, ranging it from I$0.00 to the base case value of I$2,416.33, and found that even at no cost, the ICER remained above the base case WTP threshold (Data Supplement). However, FOLFOX 6MO would be cost-effective if a higher WTP threshold of three times the GDP per capita was assumed and the administration cost was 20% of the base case value (Data Supplement).

The PSA was performed with a WTP threshold equal to the GDP per capita and accounted for uncertainty in all key model parameters. CAPOX 3MO remained optimal for 79% of iterations. The Data Supplement depicts the impact of ranging the WTP threshold between one half to three times the ZA GDP per capita on the recommended strategy. Even at the highest recommended WTP threshold, CAPOX 3MO was optimal more than 60% of the time.

### Risk-Stratified Scenario Analysis

The results of the analysis stratifying patients with stage III colon cancer by risk of colon cancer recurrence are presented in the Data Supplement. CAPOX 6MO was the optimal strategy in high-risk patients, with a lifetime cost of I$7,163.36 and utility of 6.94 DALYs averted relative to no adjuvant chemotherapy. This strategy had the highest NMB (I$83,046.12) and an ICER of I$2,136.87/DALY averted compared with CAPOX 3MO, well below the WTP threshold. In the high-risk cohort, CAPOX 3MO was on the efficiency frontier but less optimal than CAPOX 6MO despite a lower lifetime cost (I$6,075.15) because of a lower effectiveness of 6.43 DALYs averted. FOLFOX 6MO was not cost-effective in this scenario, with an ICER of I$43,040.42/DALY averted. For the low-risk scenario, the model recommended CAPOX 3MO as the optimal strategy, with a lifetime cost of I$5,138.92 and utility of 7.02 DALYs averted compared with no adjuvant chemotherapy. All other strategies were dominated for low-risk patients.

## DISCUSSION

Economic development is correlated with a rise in numerous cancer risk factors, resulting in an increase in cancer incidence and mortality in LMICs.^1^ Despite this trend, few studies have examined how health systems in low-resource settings can expand cancer treatment capacity in a cost-effective way. In this study, we used a decision-analytic Markov model to identify the cost-effective adjuvant chemotherapy strategy, compared with no adjuvant chemotherapy (surgery alone) for adult patients with stage III colon cancer in ZA public hospitals. This country- and disease-specific model provides evidence for a colon cancer treatment pathway specific to ZA public hospitals; furthermore, this analysis illustrates the merit of cost-effectiveness analyses in resource-constrained settings.

Our model predicted that for adults treated for stage III colon cancer in ZA public hospitals, all tested adjuvant chemotherapy regimens result in higher societal NMB compared with surgery alone, but that CAPOX 3MO is the cost-effective option. To our knowledge, this is the first study comparing the cost-effectiveness of specific adjuvant chemotherapy regimens for colon cancer in an LMIC setting, and one of very few that compares cancer therapeutics in LMICs.^51^ In 2012, Ginsberg et al published a sectoral cost-effectiveness analysis, which found that expanding CRC treatment generally in SSA is very-cost effective, but the methods used did not allow for country-level recommendations on cancer therapeutics.

By contrast, several studies have examined the cost-effectiveness of various adjuvant chemotherapy regimens for stage III colon cancer in HIC settings. In 2018, Aballéa et al^52^ compared the cost-effectiveness of FOLFOX 6MO versus FU/LV from a US-Medicare perspective, concluding that FOLFOX 6MO was the optimal strategy at that time (ICER: $22,804 per quality-adjusted life-year [QALY] gained, WTP: $50,000/QALY gained). Also, in 2018, the IDEA collaboration published 3-year DFS results from the pooled analysis of six RCTs comparing three versus 6 months of oxaliplatin-based adjuvant chemotherapy (FOLFOX or CAPOX) for stage III colon cancer, showing noninferiority between 3 and 6 months of CAPOX but not FOLFOX.^35^ An economic analysis using data from one of the RCTs from the IDEA collaboration showed that, from the UK health care perspective, 3 months of CAPOX or FOLFOX was cost-effective and associated with a significantly higher NMB compared with 6 months.^53^

In the base case, our model predicted that CAPOX 3MO was cost-effective in ZA public hospitals, consistent with the UK analysis that found 3 months of oxaliplatin-based therapy to be cost-effective compared with 6 months.^53^ However, our model further distinguishes between CAPOX and FOLFOX, which is important in the ZA setting as the difference in local administration costs for these two regimens is significant. We also included a risk-stratified scenario, which is absent from the UK analysis. In this analysis, CAPOX 3MO remained on the efficiency frontier for the high-risk group but the ICER for CAPOX 6MO was well within the WTP threshold, and thus, CAPOX 6MO became the optimal strategy. This is consistent with US clinical guidelines for 6 months of adjuvant chemotherapy in high-risk stage III colon cancer.^54^

Beyond our primary conclusion, our work also revealed several important drivers of cancer care costs in ZA. Most strikingly, our model highlighted the significant lifetime societal costs incurred when patients with stage III colon cancer do not receive adjuvant chemotherapy (I$9,959.24 per patient for no adjuvant chemotherapy *v* I$5,381.17 for CAPOX 3MO). The high cost of not providing adjuvant chemotherapy is driven by high rates of colon cancer recurrence and the high cost of treatment in the metastatic setting. Our model also showed the considerable cost-savings associated with oral chemotherapy, both because this decreased the number of health care visits (1 *v* 5 visits for capecitabine *v* FU/LV and 8 *v* 12 visits for CAPOX 6MO *v* FOFLOX 6MO) and because it obviated the need for costly procedures like the placement of a port-a-cath and for continuous infusion pumps.

There are several strengths and limitations of this study. The quantitative nature of a cost-effectiveness analysis limits our ability to incorporate patient voice and perspective, especially with regards to estimating the true indirect costs of treatment. Further qualitative studies are needed to understand factors other than cost that drive patient and provider decisions regarding colon cancer treatment in ZA.

With regards to our model, the most significant limitation is the assumption that patient outcomes reported in RCTs from HICs were representative of outcomes in ZA public hospitals. This assumption disregards the fact that ZA has a long history of colonialism and structural racism that has led to vast disparities in access to and quality of cancer treatment in the ZA public health sector compared with those in HICs, which likely negatively affect colon cancer survival in ZA. To address this limitation in this model as well as future cost-effectiveness models done in LMICs, international cancer RCTs must increase the enrollment of representative populations from LMICs to gather data that more accurately reflect local realties.

In the absence of such data, we used several strategies to limit the impact of this assumption on the accuracy of our model. First, we calibrated our model to DFS rather than OS from relevant RCTs. We chose this end point because OS after colon cancer recurrence depends on the number of available therapeutic options; thus, RCT OS data from HICs are likely not representative of OS in LMICs, where therapeutic options are limited. Instead, we used US-based data from 1998 to 2003 to estimate the rate of death after colon cancer recurrence in our model; these data precede the use of biologic agents for metastatic colon cancer, more closely approximating the therapeutic options currently available in ZA public hospitals. Second, to account for lower life expectancy in ZA compared with that of the participants in the RCTs, our model applied all-cause mortality rates specific to ZA. Third, our deterministic sensitivity analysis revealed that even if recurrence rates after CAPOX 3MO were 20% higher than those inputted from RCTs, this strategy remained cost-effective.

Another limitation of applying RCT data from HICs to the ZA setting is that RCT populations are majority-White, whereas the population served by ZA public hospitals is majority-Black and may experience racial disparities in access to care that lead to worse outcomes. However, data from a large, population-level study in the United States suggest that despite the existence of racial disparities across the cancer care continuum, DFS and colon cancer–specific survival between White and Black Americans are equivalent, suggesting that race-based disparities in OS among patients with colon cancer are driven by non–colon cancer deaths.^55^ Since our model is calibrated to DFS, not OS, extrapolated from relevant RCTs, and our model recommendation remains unchanged when DFS is varied substantially, we believe that the results of our model remain applicable to the majority-Black population served by ZA public hospitals.

A third limitation is that in the absence of long-term follow-up data for all treatment arms, we extrapolated Kaplan-Meier estimates of recurrence-free and DFS beyond the end points of the RCTs. Although this may have introduced uncertainty in our inputs, our model reproduced each of the curves to within 1.5% of the 5-year rates reported in the RCTs; additionally, extrapolation was only necessary for years 5-8 of our model, as the recurrence rate was assumed to be zero after 8 years (Data Supplement).^33^

In this analysis, we set the base case WTP threshold equal to the GDP per capita of ZA rather than the WHO's guideline of three times the GDP per capita, which is commonly followed in HICs, because it more closely reflects health care spending in ZA.^18,42,44,48,49^ The PSA results illustrate that the model recommendation does not change even at a WTP threshold of three times the GDP per capita.

Finally, our analysis is specific to ZA, a country at the upper end of the LMIC spectrum where multiple chemotherapy agents for colon cancer are on the country-specific Essential Medicines List and available in public hospitals; our results may not be generalizable to lower-income settings, where the availability of chemotherapy drugs, their costs, and the WTP threshold are likely to differ significantly. However, our analysis provides a framework for how to build country-specific decision-analytic models and how cost-effectiveness analyses can be used to inform oncology treatment and policy decisions in LMIC settings.

In conclusion, using a decision-analytic Markov model developed for ZA public hospitals, we show that adjuvant chemotherapy with CAPOX for 3 months is cost-effective for treating patients with stage III colon cancer and should be the preferred regimen. Our model predicts substantial benefits in terms of survival, quality of life, and total health care and societal costs when a health system in an LMIC invests in funding cost-effective adjuvant chemotherapy. Performing similar cost-effectiveness analyses in other LMICs may provide critical setting-specific evidence to guide rational cancer treatment pathways.

## Data Availability

The data generated during the current study are available from the corresponding author by request.
